# Isolated Rat Cardiomyocytes Differ in Their Sensitivity to the Action of Heterodimeric Phospholipase A2 from the Venom of the Viper *Vipera nikolskii*

**DOI:** 10.3390/ijms27209353

**Published:** 2026-10-21

**Authors:** Miliausha Galimova, Oleg Pimenov, Ilia Teplov, Alexey Berezhnov, Vladislav Starkov, Victor Tsetlin, Yuri Utkin

**Affiliations:** 1 Institute of Theoretical and Experimental Biophysics, Russian Academy of Sciences, Pushchino 142290, Russia; mgalimova@mail.ru (M.G.); polegiteb@gmail.com (O.P.); 2 Institute of Cell Biophysics, Federal Research Center “Pushchino Scientific Center of Biological Research”, Pushchino Branch, Russian Academy of Sciences, Pushchino 142290, Russia; teplov_iy@pbsras.rus (I.T.); alexbereg56@gmail.com (A.B.); 3 Shemyakin-Ovchinnikov Institute of Bioorganic Chemistry, Russian Academy of Sciences, Moscow 117997, Russia; vladislavstarkov7398@gmail.com (V.S.); victortsetlin3f@gmail.com (V.T.)

**Keywords:** cardiomyocyte, cytosolic calcium, electrical stimulation, fluorescence, hypercontracture, phospholipase A2, snake venom

## Abstract

Snake venom phospholipase A2 (PLA2) affect, among others, the cardiovascular system; however, data on their effect on isolated cardiomyocytes are practically absent. In this work, we investigated the influence of HDP-1 and HDP-2, heterodimeric PLA2s from the venom of viper *Vipera nikolskii*, on isolated rat cardiomyocytes. Cardiomyocytes were stained with fluorescent probes and fluorescence was measured using Cell Observer fluorescent station. After PLA2 application, there was a prolonged lag phase with no changes in cell morphology or probe signal, followed by fluctuations in the cytosolic calcium level and cell hypercontracture with subsequent disruption of plasma membrane. In individual cardiomyocytes, time from the toxin addition until the hypercontracture varied by more than two-fold. In experiments with electrical stimulation, at HDP-2 application, a “pause” in contractions was observed, characterized by a decrease in the amplitude of the fluorescent response. Then, the contraction rhythm recovered, and later, hypercontracture was observed followed by cell destruction. Depending on times to these events, all cardiomyocytes studied were classified into two groups with fast and slow response to PLA2 action. The observed differences in the sensitivity of cardiomyocytes may be explained by unequal concentrations of intracellular calcium due to the different states of the cells at isolation.

## 1. Introduction

Cardiovascular system is one or the main targets for animal venoms, and venoms of different snake species produce cardiotoxic effects [1,2,3]. Thus, the venom of the Chocoan bushmaster *Lachesis acrochorda* increased the frequency of spontaneous contractions in isolated rat right atrium preparations dose-dependently in the range 1–1000 μg/mL [2]. A similar effect was observed for the venom of the Western diamondback rattlesnake *Crotalus atrox* [4]. It was found that this venom enhanced contractility and frequency of contractions in ex vivo Langendorff heart model. The opposite effect was observed for the venom of nose-horned viper *Vipera ammodytes ammodytes* on isolated rat heart. This venom produced dose-dependent depression of contractility and coronary flow. Biphasic effects on the heart rate were observed with transient increase in the frequency followed by its significant decrease. Irreversible asystolic cardiac arrest occurred at a venom dose of 150 μg/mL [5]. In a similar way in ex vivo Langendorff heart model, the venom of red spitting cobra *Naja pallida* decreased contractile force [4]. It is well-known that cobra venoms comprise cytotoxins from the three-finger toxin family affecting the heart function [6] and these toxins often are called cardiotoxins. Earlier we have shown that cardiotoxins produced a complete suppression of heart papillary muscle contraction [7] and in isolated rat cardiomyocytes these toxins caused the development of irreversible hypercontracture due to the calcium overload [8]. However, the venoms of Viperidae snakes for which cardiotoxic effects were shown do not contain cardiotoxins, and other toxin(s) causing adverse cardiac effects may be present in these venoms. And this is indeed true. Thus, in isolated rat right atria, concentration-dependent contractures were produced by the venom of pit viper *Bothrops jararacussu* and this effect was not abolished by washing [9]. The venom-induced contracture was canceled by the treatment of venom with p-bromophenacyl bromide, a phospholipase A2 (PLA2) inhibitor. These results indicated the participation of PLA2 in the adverse effect produced by the venom in rat atria [9].

A detailed study of snake venoms that affect heart function has led to the identification of several toxins that exhibit cardiotoxicity [10], and these toxins include PLA2s, which form one of the most extensive families of snake toxins. PLA2s from a variety of snake species were shown to influence cardiac activity. Thus, cardiotoxic effects were manifested by PLA2s from cobra *Naja nigricollis* venom [11], king cobra *Ophiophagus hannah* [12], Northern cottonmouth *Agkistrodon piscivorus piscivorus* [13], and South American rattlesnake *C. durissus terrificus* [14]. Recently, we found that in the rat papillary muscle, the heterodimeric PLA2 HDP-1 from the viper *V. nikolskii* over a wide range of concentrations produced a long-term, stable, positive inotropic effect, which did not turn into contracture [15].

PLA2s ubiquitously present in snake venoms belong to the so-called secreted PLA2s. These PLA2s are typically small proteins possessing a molecular weight in the range of 13–15 kDa [16,17]. Secreted PLA2 are divided into 10 groups and 18 subgroups [18], PLA2s from snake venoms belonging to groups I and II [16]. This classification scheme fundamentally relies on four major criteria: phylogenetic origin/snake family, disulfide bond arrangements, secondary structural motifs, and active site catalytic residues/mutations. Although most of PLA2s are single-chain toxins with six or seven disulfide bridges, enzymes that include several subunits are also known. Thus, some PLA2s are composed of two homologous subunits linked non-covalently, with at least one of the subunits possessing phospholipolytic activity. For example, such heterodimers are present in venoms of snakes from the *Vipera* genus. Vipoxin from *V. ammodytes ammodytes* [19] as well as HDP-1 and HDP-2 from *V. nikolskii* [20] should be mentioned. In HDP-1 and HDP-2, both enzymatically active and inactive subunits consist of 122 amino acid residues which include 14 cysteine residues forming seven disulfide bonds. In the putative active site of the inactive subunit, a histidine residue is replaced by a glutamine residue, which leads to the loss of enzymatic activity. In HDP-1 and HDP-2, the inactive subunits are the same and enzymatically active subunits differ only by two amino acid residues. Lysine 100 in HDP-2 is replaced with glutamine in HDP-1 and arginine 108 in HDP-2 with lysine in HDP-1, which makes HDP-1 less basic than HDP-2. Although these changes are outside the active center, they result in small differences in activity between these two PLA2s. HDP-1 possesses higher enzymatic activity than HDP-2, while the effects of HDP-2 on the neuromuscular transmission is more pronounced [20]. PLA2s comprised more than two subunits are also known. Thus, trimers are represented by taipoxin from *Oxyuranus scutellatus* and paradoxin from *O. microlepidotus* [21], and textilotoxin produced by *Pseudonaja textilis textilis* is a pentamer [22].

The observed effects of phospholipases may be due to their direct action on cardiomyocytes. However, the data about PLA2 effects on cardiomyocytes is scarce. Thus, crotoxin, a heterodimeric PLA2 from the South American rattlesnake *C. durissus terrificus* affected electrical activity of neonatal rat cardiomyocytes and increase in CTX concentration to 3–4 microM completely blocked cardiomyocytes beating [23]. It was shown also that devoid of enzymatic activity Lys49 PLA2 homologs from *B. asper* venom induced rapid hypercontraction of intact rat cardiomyocytes with a simultaneous increase in the concentration of cytosolic calcium and with cell membrane damage [24].

The aim of this study was to investigate the difference in the effects of PLA2s on mechanically quiescent (non-contracting) and contracting cardiomyocytes. Freshly isolated healthy adult cardiomyocytes from rat hearts and heterodimeric PLA2s HDP-1 and HDP-2 from *V. nikolskii* venom were used for this purpose. It was established that, in both mechanically quiescent and contracting cardiomyocytes, exposure to HDP-2 induced an increase in cytosolic calcium levels followed by hypercontracture and cell destruction. The time to cell destruction depended on HDP-2 concentration. The contracting cardiomyocytes were found to be more susceptible to the toxic effects of PLA2. Moreover, isolated individual cardiomyocytes demonstrated different sensitivity to PLA2 action. Depending on the time until cell destruction under the influence of HDP-2, that is, sensitivity to its action, contracting cardiomyocytes can be divided into two groups, one of which is more sensitive and the other less sensitive to HDP-2. This is the first indication of the existence of such groups of cardiomyocytes.

## 2. Results

The adverse effects of various substances on cardiomyocytes are often associated with impaired regulation of intracellular calcium levels and calcium overload of the cell. To investigate the effects of PLA2s on cardiomyocytes, we monitored intracellular Ca^2+^ concentration using a fluorescence measurement with a ratiometric fluorescent dye Fura-2, which binds to free intracellular calcium ions. The isolated cardiomyocytes were loaded with Fura-2 AM, which once internalized is transformed into Fura-2 active form by cellular esterases and binds to free intracellular calcium ions, and then different concentrations of HDP-1 or HDP-2 were added. The time elapsed from the moment of toxin addition until the onset of hypercontracture (TtH), when the width of the cell exceeds its length, was also recorded.

**Figure 1:**
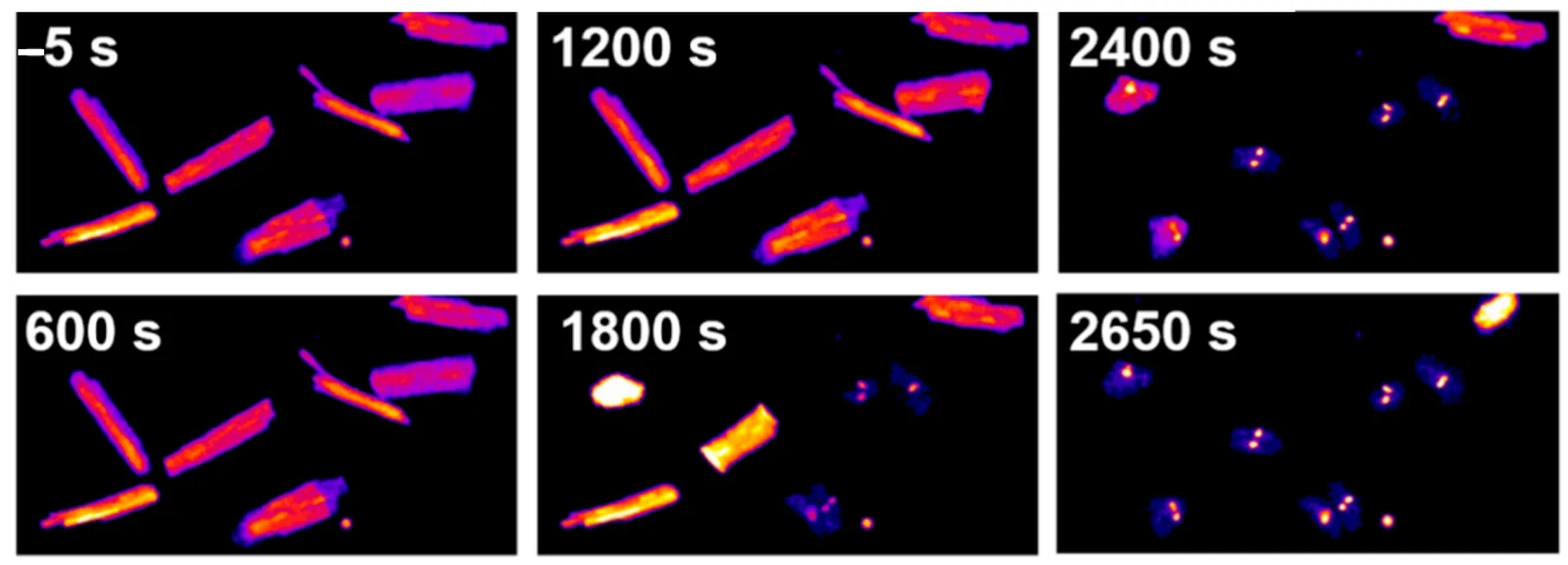
Fluorescence changes in individual Fura-2-loaded cardiomyocytes after application of 10 μM HDP-2. Increase in [Ca^2+^]i shifts the color to yellow. Excitation at 340 nm. Pseudo color. The time elapsed from the moment of toxin addition is shown on each panel. Part of the observed cells is shown.

Individual cardiomyocytes were observed under the microscope. The results of the experiment with 10 μM HDP-2 (n = 14) added are presented in Figure 1. After application of 10 μM HDP-2, there was a prolonged lag phase with no noticeable changes in cell morphology or fluorescent probe signal. Subsequently, some cells experience fluctuations in cytosolic calcium ion concentration ([Ca^2+^]i), followed by a sharp increase in [Ca^2+^]i and spontaneous contractions (Video S1). Then the onset of hypercontracture occurs, characterized by a sharp, 2–3 times decrease in the length of the cardiomyocyte and the transition of its shape to an almost spherical one (Figure 1), followed by the decrease in fluorescence level due to the loss of the fluorescent probe, indicating disruption of the plasma membrane’s integrity (Video S1).

Of the 14 cells in the experiment, the minimum TtH was 1080 s; the maximum was 2630 s (Figure 1). The average was 1922 +/− 488 sec (Mean +/− SD). In the studied set of cardiomyocytes, TtH varied by more than two times.

After incubation for 40 min at a concentration of 1 μM, neither HDP-1 (n = 27 cells) nor HDP-2 (n = 18 cells) produced changes in morphology and [Ca^2+^]i in the treated cardiomyocytes.

**Figure 2:**
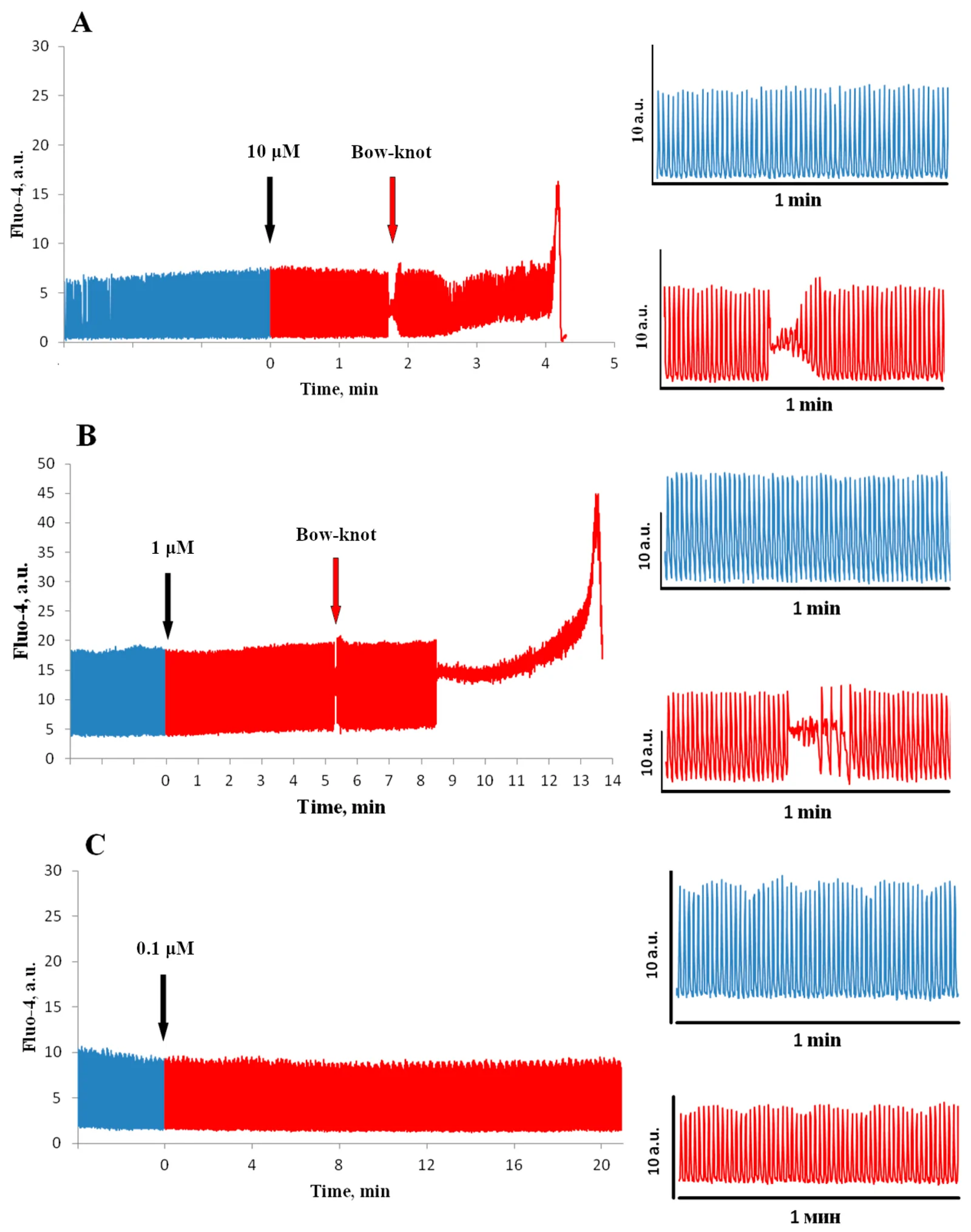
Representative traces of fluorescent dye responses in isolated cardiomyocytes stimulated by electrical field at 1 Hz in control (blue) and under treatment with HDP-2 (red) at following concentrations: 10 μM (**A**), 1 μM (**B**) and 0.1 μM (**C**). a.u.—arbitrary unit.

To study the effects of PLA2s on functioning cardiomyocytes, electrical stimulation was employed. The stimulation was done by a glass microelectrode placed directly to the selected cardiomyocyte; rectangular electrical impulses with a frequency of 1 Hz were applied. In the experiments under conditions of electrical stimulation, after recording control values of the fluorescent response, HDP-2 was added, and changes in the fluorescence were recorded until cell death. Since HDP-2 had a long-term effect in experiments recording spontaneous calcium waves only at a concentration of 10 μM, we began our experiments under conditions of electrical stimulation at this concentration. On isolated cardiomyocytes, control values of the fluorescence response were recorded for the first 3–5 min; then HDP-2 was added to the medium at concentrations of 10 μM, and changes in the fluorescence response were further recorded. The lower HDP-2 concentrations of 1 and 0.1 μM were also studied. In the effect of HDP-2, several stages, the duration of which depends on the concentration of HDP-2, can be distinguished. Thus, approximately at 2 min after application of 10 μM HDP-2, the regular fluorescent responses were disturbed, demonstrating the increase in intracellular calcium levels and lowering of individual fluorescent spikes for a short time (about 10 sec) with subsequent restoration of regular activity; this region is called as “bow-knot” (Figure 2A).

However, continued exposure of the cardiomyocites to HDP-2 results in increased destructive effects, resulting in an increasing rise in intracellular calcium level with subsequent cell death. This is clearly shown in Figure 2A. At about 1.5 min after the observed activity restoration, a slow increase in cytoplasmic calcium with slight alteration in individual spike amplitude was seen (Figure 2A). Finally, the cell death manifested by intensive spike followed by sharp drop in fluorescence occurred at about 4 min of HDP-2 action (Figure 2A).

Application of HDP-2 at 1 μM exhibited different dynamics of fluorescent responses, especially at later stage. The beginning of each stage was shifted to longer times. Thus, the first increase in intracellular calcium levels and lowering of individual fluorescent spikes for a short time (“bow-knot” region) occurred at about 5 min of HDP-2 action (Figure 2B) compared to 2 min at 10 μM HDP-2. Then the amplitude of the fluorescent responses was fully restored and this continued for another 8 min (Figure 2B). It should be noted that the further dynamics of fluorescence changes at application of 1 μM HDP-2 (Figure 2B) was dramatically different from that observed at 10 μM HDP-2. Cytosolic calcium levels were raised quickly with simultaneous lowering of individual peaks amplitude—cell twitched for about 5 min until its death. HDP-2 action on fluorescent dye responses exhibited dose dependency in quite narrow range, increasing the times of characteristic phases with decreasing HDP-2 concentration down to no reaction at 0.1 μM (Figure 2C).

Since HDP-2 at 1μM concentration produced the pronounced effect on fluorescent dye responses, we used HDP-1 at the same concentration. It has been shown that HDP-1 (1 μM) was not able to modify intracellular calcium handling and therefore did not affect fluorescent responses; the traces look the same as for HDP-2 action at 0.1 μM concentration (Figure 2C).

**Figure 3:**
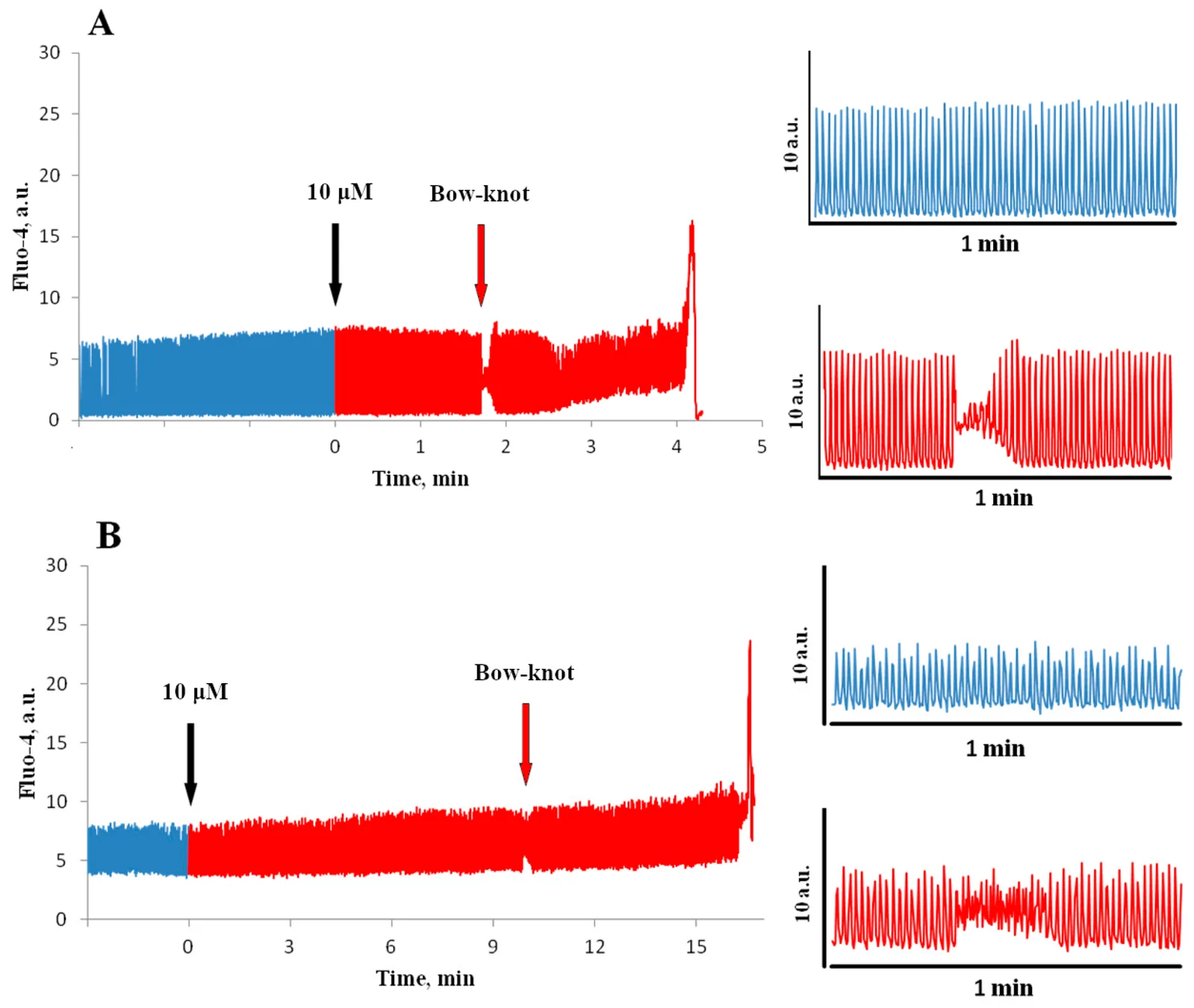
Comparison of representative traces of fluorescent dye responses in isolated cardiomyocytes of Group I (**A**) and Group II (**B**) to electrical stimulation at 1 Hz in control (blue) and under treatment of 10 μM HDP-2 (red). a.u.—arbitrary unit.

It should be noted that in individual cardiomyocytes, the times to bow-knot and cell death differ even if HDP-2 was applied at the same concentration. Cardiomyocytes can be divided into two distinct groups based on their response to electrical stimuli: Group I, which is characterized by a rapid response to HDP-2, and Group II, which shows a slow response to HDP-2 (Figure 3). This may be due to differences in initial physiological state of individual cardiomyocytes in primary culture. Thus, in Group II, cell death was observed approximately 17 min after applying 10 μM HDP-2 (Figure 3B), compared to approximately 4 min in Group I (Figure 3A).

**Figure 4:**
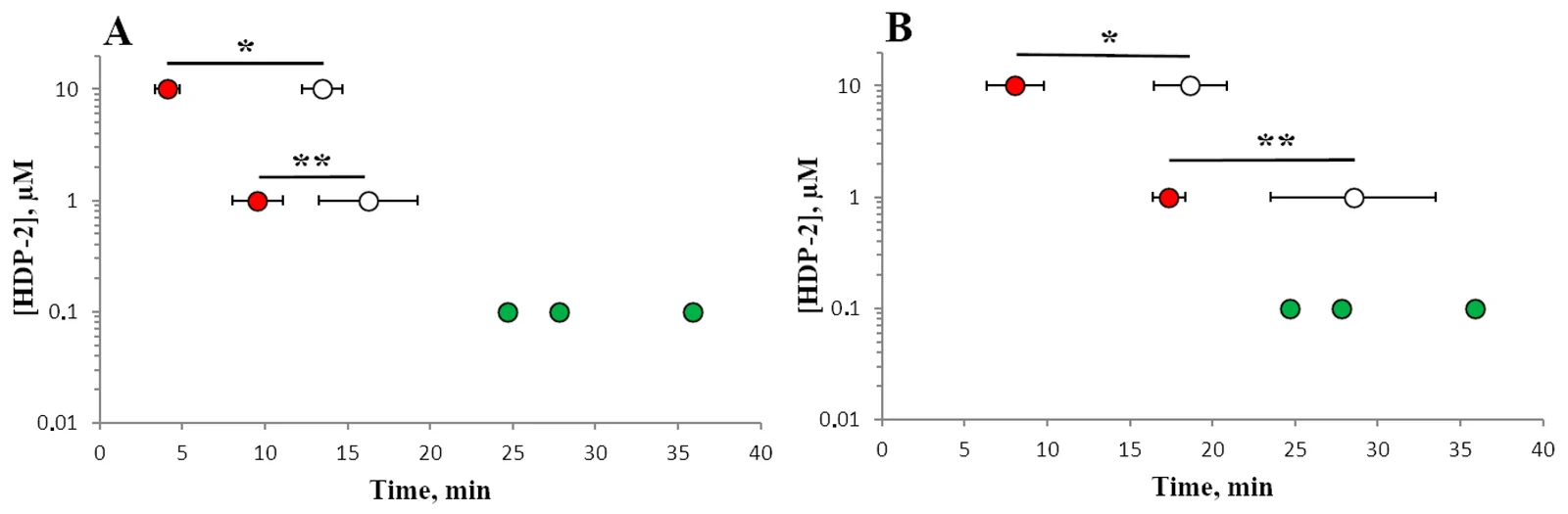
Concentration dependence of HDP-2 action on fluorescent dye responses in isolated cardiomyocytes of Group I (red) compared to Group II (white). Green circles represent individual cells, which survived during application of 0.1 µM HDP-2. They indicate the time during which the signal was recorded. Characteristic times of “bow-knot” region (**A**) and of cell death point (**B**) are represented as mean ± standard deviation. * *p* < 0.005; ** *p* < 0.05.

Moreover, those differences may alter dynamics of fluorescent responses to HDP-2 in cardiomyocytes Group II, shifting observed phases in time and thus prolonging whole trace, even if visible contractile responses of cardiomyocytes to applied electrical stimulus seemed to be normal (Table 1, Figure 4).

## 3. Discussion

As it was mentioned in introduction, the venom of viper *Vipera nikolskii* comprises two heterodimeric PLA2—HDP-1 and HDP-2 [20]. These toxins differ in their amino acid sequences by only two amino acid residues at positions 100 and 108 which results in only small differences in their biological properties [20]. Recently, we have shown that HDP-1 and its enzymatically active subunit HDP-1P affected the functional activity of the rat heart, producing an arrhythmia [25]. Together with earlier mentioned work about the HDP-1 effect on rat papillary muscle [15] these may indicate the influence of this PLA2 on cardiomyocites. In this work we studied the effect of HDP-1 and HDP-2, another heterodimeric PLA2 from *V. nikolskii*, on the rat heart cardiomyocytes.

For this purpose, freshly isolated non-beating adult rat cardiomyocytes were used. These cardiomyocytes, without stimulation, were treated with different concentrations of PLA2s. While HDP-1 and HDP-2 at concentrations of 1 µM produced no effects, HDP-2 at 10 µM induced cell hypercontructures followed by death. This was the consequence of the strong increase in [Ca^2+^]i induced by HDP-2 action on the cell membrane. The shape of the cells was also strongly changed due to disruption of the integrity of plasma membrane [26,27]. The development of irreversible hypercontracture, which previously was observed under the action of different toxic agents [28,29], is a result of the calcium overload.

Subsequent experiments using electrical stimulation demonstrate reduction in the characteristic times of cell death in response to HDP-2 application, thus indicating strong dependence of HDP-2 targeting and/or embedding to plasma membrane of the cardiomyocytes. Moreover, under stimulation conditions we observed a so-called “bow-knot” region, that indicates onset of the HDP-2 toxic effect and its duration may characterize intracellular capacity to overcome this. Nevertheless, the continuous HDP-2 embedding overcomes intracellular restoration mechanisms, leading to intracellular calcium accumulation and, finally, to the cell death. Our experiments revealed that cardiomyocytes population can be divided into two distinct groups based on their response to electrical stimuli: Group I, which is characterized by a rapid response to HDP-2, and Group II, which shows a slow response to HDP-2. This may be attributed to differences in the physiological state of individual cardiomyocytes in the primary culture, which affects the duration of the characteristic phases of HDP-2 action. It should be mentioned that earlier works have shown some heterogeneity in mechanical and electrical properties of single cardiomyocytes isolated from the left ventricle [30,31,32].

The lethal effects on isolated cardiomyocytes were earlier observed for the catalytically inactive Lys49 PLA2 homolog Myotoxin II (Mt-II) from *Bothrops asper* venom; Mt-II is characterized by having a Lys residue in place of the canonical Asp at position 49 of the amino acid sequence [24]. On intact electrically stimulated cardiomyocytes obtained from the ventricles of adult male Lewis rats, after a short period, Mt-II induced a permanent hypercontracture. The time to achieve the hypercontracture was dependent on the Mt-II concentration, being 17.3 min and 2.7 min for 0.9 and 3.6 μM, respectively. These times are shorter than those observed in our experiments. Thus, even for fast-responding Group I, time to cell death, which practically coincide with TtH, was 13.46 min at 10 μM HDP-2. The lower HDP-2 activity may be explained by differences in the chemical structures of the two toxins. Thus, Mt-II, being catalytically inactive Lys49 PLA2 homolog, is a monomeric myotoxin with quite strong cytotoxicity [33], while HDP-2 is dimeric PLA2 with much lower cytotoxicity [34].

Interestingly, while HDP-2 at 1μM concentration produced the pronounced effect on fluorescent dye responses, HDP-1 at the same concentration did not affect fluorescent responses. Although the amino acid sequences of HDP-1 and HDP-2 differ in only two positions, this appears to be sufficient to reduce the toxicity of HDP-1 towards cardiomyocytes. It is quite possible that replacing lysine 100 in HDP-2 with glutamine and arginine 108 in HDP-2 with lysine in HDP-1, which leads to a decrease in the basicity of HDP-1, reduces the effectiveness of its interaction with the phospholipids of the cardiomyocyte membrane.

So, HDP-2 produced a fairly slow calcium influx in cardiomyocytes followed by contracture, whereas Mt-II induced rapid plasma membrane disruption, calcium influx and increments in cytosolic calcium concentration leading to hypercontracture [24].

Typically, isolated healthy adult mammalian ventricular cardiomyocytes do not spontaneously beat outside the body; instead, they remain non-beating and rod-shaped unless artificially stimulated by electrical pacing. Some physiological processes occur differently in non-beating and beating cardiomyocytes. To study the effects of PLA2s on functioning cardiomyocytes, they were stimulated electrically. Following HDP-2 application after a certain period of time, which depended on the PLA2 concentration and stimulation intensity, we observed a transient decrease in the intensity of fluorescent responses to stimulation. This occurred for approximately 10 s, followed by a recovery in the intensity of fluorescent signals—the bow-knot region. The normal responses continued for a certain time more and ended with cell death.

It should be noted that in cardiomyocytes, the relationship between [Ca^2+^]i levels and the amplitude of individual fluorescent spikes is characterized by a feedback mechanism where sustained increases in baseline calcium can lead to a reduction in individual spike magnitude. After addition of HDP-2 to cardiomyocytes in the chamber, its action results in the degradation of outer membrane lipids which can lead to increased membrane permeability and disruption of the intracellular ionic balance, in particular, to an increase in cytoplasmic calcium levels [35]. This leads to a decrease in the calcium gradient and, consequently, to a decrease in the magnitude of the fluorescent response and, accordingly, to a reduction in the amplitude of a single spike which is seen in the bow-knot region (Figure 2 and Figure 3). To explain the further events, we can put forward the following hypothesis. In response to the cytotoxic effect of HDP-2, compensatory mechanisms aimed at restoring the integrity of the plasma membrane may be triggered in the cell, which can lead to a temporary restoration of the normal fluorescent response. Continued exposure of the cardiomyocites to HDP-2 may result in increased destructive effects, and as intracellular reserves are depleted, an increasing rise in intracellular calcium level with subsequent cell death. However, this mechanism requires further experimental validation. As far as we know, the effect of PLA2 on cardiomyocytes, which can be called a bow-knot, was discovered by us for the first time.

The effects of toxins possessing phospholipolytic activity on spontaneously contracting neonatal rat cardiomyocytes have been previously investigated [23,36]. Such data were obtained for taicatoxin—an oligomeric complex of three non-covalently linked polypeptides, one of which exhibits phospholipolytic activity—and crotoxin, which consists of two subunits, one being a phospholipase A2. For instance, taicatoxin induced transient yet severe arrhythmias and prolonged alterations in intercellular electrical coupling in spontaneously contracting cardiomyocytes [36]. Taicatoxin’s effects were observed at concentrations of 0.1 and 1 µM, and the authors suggested they were consistent with the specific blockade of L-type voltage-gated calcium channels [36]. Later, it was found that crotoxin significantly enhanced currents through L-type Ca^2+^ channels in spontaneously contracting cardiomyocytes [23]. At a concentration of 2 µM, crotoxin doubled the Ca^2+^ channel currents while causing a slight, short-lived increase in the contraction rate of some cardiomyocytes. At a concentration of 1 µM, it did not affect the contraction rate, whereas at concentrations of 3–4 µM, the contractile activity of the majority of cells was suppressed [23]. Crotoxin also affected the action potential; however, this effect was observed at a concentration of 6 µM and was accompanied by a decrease in the amplitude and frequency of the current spikes. It should be noted that no cardiomyocyte death was observed upon exposure to taicatoxin or crotoxin. This differs significantly from the action of HDP-2, which caused the death of electrically stimulated cardiomyocytes at a concentration of 1 µM. This difference can likely be attributed to the structural characteristics of these toxins. Interestingly, we found that the cardiomyocytes studied in this work can be divided in two groups according to their responses to the HDP-2 application. One group is fairly sensitive to HDP-2 action in which its application at 10 µM to stimulated cardiomyocytes resulted in the death of the cells in about 13 min and of another group less sensitive with time to the cells’ death of about 19 min. These differences may be explained by different factors including differences in the initial state of the cells before isolation or the differences that may arise during the isolation and/or incubation of the cells. However, the exact explanation for why cardiac myocytes are clearly divided into two groups requires further research.

It should be noted that, like all PLA2s, HDP-2 exhibits phospholipolytic activity. A crucial question concerns the role this phospholipolytic activity plays in the effects we have observed. HDP-2 is a dimeric PLA2 composed of an enzymatically active subunit and an inactive one; these subunits can readily be obtained in isolated form. Investigating the effects of the individual subunits on isolated cardiomyocytes could clarify the role of phospholipolytic activity in the observed phenomena. This constitutes the objective of future research into the effects of HDP-2 on cardiomyocytes.

## 4. Materials and Methods

### 4.1. Materials

Fura-2 AM was from Invitrogen (cat. no. F1221, Eugene, OR, USA). Coverslips Menzel-Gläser Ø25 mm #1 were from Thermo Scientific (Darmstadt, Germany). All salts and other reagents were purchased from PanEco (Moscow, Russia).

HDP-1 and HDP-2 were prepared from V. nikolskii venom obtained from the snakes kept in captivity as described [20]. In brief, the milked venom was dried over anhydrous CaCl_2_ and stored at −20 °C until used. For the isolation of toxins, an aliquot of the venom was dissolved in 5 mM solution of ammonium acetate (pH 7.5) and applied on cation exchange column TSKgel CM-3SW (7.5 × 75 mm, Tosoh Bioscience GmbH, Griesheim, Germany). The column was eluted with an ammonium acetate gradient from 5 to 700 mM (pH 7.5) in 140 min at a flow rate of 1 mL/min. Fractions containing HDP-1 and HDP-2 were collected, freeze-dried and used for further studies. The purity of HDP-1 and HDP-2 was confirmed by analytical ion-exchange chromatography and structure was proved by mass spectrometry.

This study did not involve endangered or protected species and was performed in accordance with Directive 2010/63/EU of the European Parliament. All animal procedures were approved by the Ethics Committee at the Institute of Theoretical and Experimental Biophysics, RAS, Russia (Protocol No. 12/2023 dated 8 February 2023). Adult male Wistar rats (240–300 g body weight) were used for the experiments.

### 4.2. Cardiomyocyte Preparation

Cardiomyocytes were prepared essentially as described [8]. In brief, cardiomyocytes were isolated by enzymatic dissociation from the hearts of Wistar rats. To isolate more cardiomyocytes, the left ventricles accounting for approximately 80% of the total mass of the hearts were used. After animal decapitation, the heart was extracted and retrogradely perfused for 3–5 min with DMEM + 10 mM HEPES medium (pH 7.25) (Merck KGaA, Darmstadt, Germany). After stabilization of cardiac contractions, perfusion was continued with a basic medium containing (in mM): NaCl, 80; KCl, 10; KH_2_PO_4_, 1.2; MgSO_4_, 5; glucose, 20; taurine, 50; HEPES, 10; L-arginine, 1, pH 7.25, supplemented with 2.5 mM EGTA until cardiac arrest. Then, the basic medium was supplemented with 0.2 mg/mL of protease Type E (SERVA Electrophoresis GmbH, Heidelberg, Germany), 1 mg/mL bovine serum albumin (fraction V, Merck KGaA, Darmstadt, Germany), and 140 μM CaCl_2_. After 10 min, the left ventricle was separated from the atria and right ventricle and cut into small fragments in a basic medium enriched with 200 μM CaCl_2_. Single cells were then isolated by stirring in the basic medium supplemented with protease Type E (SERVA) and collagenase A (0.25 mg/mL; Worthington Biochemical Corp., Lakewood, NJ, USA) at 37 °C. The aliquots were removed at 20 min intervals until the tissue was entirely digested. Isolated cardiomyocytes were precipitated by centrifugation (800 rpm, 1 min), washed twice, and stored in basic medium containing 200 μM CaCl_2_ at room temperature. Only rod-shaped cardiomyocytes with clear striations were used.

### 4.3. Dye Loading

For staining with a fluorescent calcium-sensitive ratiometric probe Fura-2, the cells were placed in the center of a round coverslip with a diameter of 25 mm and a thickness of 0.17 mm in a drop of 200 μL; then the cells were allowed to precipitate for 2 min. Using a pipette, the medium was replaced with 200 µL of buffer containing 5 µM Fura-2 AM. Cells were incubated for 40 min at 30 °C in the dark. Then, the medium was replaced with buffer without dye with a pipette, a coverslip was mounted in a special measuring cell for an inverted microscope, and the cells were washed again. The volume of the medium was adjusted to 1 mL and the measuring cell was placed on the microscope stage for the experiment.

### 4.4. Fluorescence Microscopy

The fluorescence in cardiomyocytes was measured using the Cell Observer fluorescent station with a 21HE filter set based on an AxioVert 200 M motorized inverted microscope (Carl Zeiss AG, Oberkochen, Germany) equipped with a 10× PlanApochromat objective, an Orca-Flash R2 monochrome camera (Hamamatsu Photonics K.K., Iwata City, Japan), and a system for a high-speed change in excitatory filters Ludl MAC 5000 (Ludl Electronic Products, Hawthorne, NY, USA). Isolated cardiomyocytes were placed on 25 mm round coverslips and stained with Fura-2 for 40 min in low calcium Hanks’ balanced salt solution (HBSS) with the following composition (in mM): 138 NaCl, 1.3 CaCl_2_, 0.4 MgSO_4_, 0.5 MgCl_2_, 5.3 KCl, 0.45 KH_2_PO_4_, 4 NaHCO_3_, 0.3 Na_2_HPO_4_, 10 glucose, 20 HEPES (pH 7.35 at 29–30 °C was adjusted with NaOH), followed by a wash. Cardiomyocytes were stained with fluorescent probes Fura-2 or Fluo-4.

### 4.5. Reagents Application

To ensure uniform distribution of the added PLA2 (HDP-1 or HDP-2) in the cell incubation medium, the substances were applied as follows: from 1 mL of HBSS in the measuring cell, half was taken, and a solution of PLA2 with double concentration was prepared in a separate microtube. After that, the PLA2 solution was returned to the measuring cell and gently mixed twice with a micropipette. In the control experiments, no noticeable effect of the taking and mixing of the medium on the Fura-2 or Fluo-4 signal was observed.

### 4.6. Image Processing

The two-channel series of images was processed in Image J/FiJi 2.9.0 (NIH, Bethesda, MD, USA). After determining and subtracting the background signal, the images were smoothed with a simple anti-aliasing filter with a 3 × 3 px grid and the ratio of the data for the 340 nm channel to those of the 380 nm channel, in 32-bit format, was calculated. Next, ROIs (regions of interest) corresponding to the position of the cardiomyocyte were selected in order to capture only the area of the cell, regardless of its movement during the contraction. A table of the ratio of Fura-2 or Fluo-4 fluorescence signals at 340 and 380 nm excitation versus time was calculated. In some cases, to determine the moment of destruction of the integrity of the plasmalemma by the outflow of the fluorescent probe, the channel for registering the calcium-bound form of the dye (channel 340) was used. Origin 2016 (OriginLab Corporation, Northampton, MA, USA) and GraphPad Prism 8 (GraphPad Software Inc., Boston, MA, USA) were used to plot the graphs.

### 4.7. Electrical Stimulation Experiments

For the experiments under conditions of electrical stimulation, isolated cardiocytes were loaded with dye by adding 5 μM Fluo-4 for 40 min, followed by three washes of the dye from the external medium with HBSS, pH = 7.35. The change in the response of the fluorescent probe to the addition of HDP-2 to the medium was recorded using an Axiovert 200M inverted microscope (Carl Zeiss, Oberkochen, Germany) and an ORCA-Flash 2.8 Digital CMOS high-speed digital camera (Hamamatsu Photonics, Iwata City, Japan). Changes in fluorescence were observed under electrical stimulation conditions by placing a glass microelectrode directly to the selected cardiomyocyte and applying a rectangular electrical stimulus with a frequency of 1 Hz. The stimulus amplitude was selected individually upon achieving a full contractile act, which was evaluated visually.

### 4.8. Data Analysis and Statistics

Paired Student’s *t*-test was used for the comparison of two groups for each HDP-2 concentration. The difference was considered statistically significant at *p* < 0.05. All data are presented as mean ± standard deviation (SD), *n* = 4–6.

## 5. Conclusions

The effects of HDP-1 and HDP-2, heterodimeric PLA2s from the venom of viper *V. nikolskii*, on isolated rat cardiomyocytes were studied using fluorescence methods. It was found that application of 10 μM HDP-2 to cardiomyocytes resulted in a prolonged lag phase with no changes in cell morphology or probe signal. Then, fluctuations in the cytosolic calcium level ([Ca^2+^]i) were observed, followed by a sharp increase in [Ca^2+^]i and hypercontracture, with subsequent disruption of the plasma membrane and cell death. In individual cardiomyocytes, the time from the toxin addition until the hypercontracture varied by more than two times. In the experiments under conditions of electrical stimulation, after recording control values of the fluorescent response, HDP-2 (0.1–10 µM) was added and changes in the fluorescence were recorded until cell death. At HDP-2 concentrations of 1 and 10 µM, a “pause” in contractions was observed, characterized by an increase in intracellular calcium and decrease in the amplitude of the fluorescent response. Then, the rhythm recovered, and later, hypercontracture was observed followed by cell destruction. The times to these events depended on the HDP-2 concentration and the efficiency of stimulation. No effects on cardiomyocytes were observed upon application of 1 µM HDP-1. Depending on the times to these events, all cardiomyocytes studied were classified into two groups with fast and slow response to PLA2 action. The differences in the sensitivity of cardiomyocytes that we discovered may be explained by different concentrations of intracellular calcium due to the different state of the cells at the time of isolation.

## Figures and Tables

**Table 1:** Differences between cardiomyocyte groups in response to HDP-2 application.

Event Point	HDP-2 Concentration	Time to Event, min				*p*
		Group I		Group II		
		Mean	SD	Mean	SD	
“Bow-knot”	10 µM	4.08	0.77	8.05	1.72	0.0008
	1 µM	9.56	1.51	17.37	0.98	0.02
Cell death	10 µM	13.46	1.24	18.64	2.20	0.009
	1 µM	16.24	2.98	28.52	5.00	0.02
No event	0.1 µM	-	-	-	-	-

## Data Availability

The original data obtained in this work are included in the article. Further inquiries can be directed to the corresponding author.

## Supplementary Materials

The following supporting information can be downloaded at: https://www.mdpi.com/article/10.3390/ijms27209353/s1.

## Abbreviations

The following abbreviations are used in this manuscript:

PLA2	Phospholipase A2
TtH	The time elapsed from the moment of toxin addition until the onset of hypercontracture
[Ca^2+^]i	Cytosolic calcium ion concentration
